# Prevention and Management of Driveline Infections in Ventricular Assist Device Patients: The DESTINE 2.0 Staging Proposal and Expert Recommendations

**DOI:** 10.1111/aor.70237

**Published:** 2026-09-09

**Authors:** Alexander M. Bernhardt, Volker Lauenroth, Florian Mueller, Friedrich Kaufmann, Katharina Homann, Martina Socha, Thomas Schlöglhofer, the Driveline Expert STagINg and carE (DESTINE) study group

**Affiliations:** ^1^ Department of Cardiac Surgery, University Heart and Vascular Center Hamburg University Hospital Hamburg‐Eppendorf Hamburg Germany; ^2^ Department for Thoracic‐ and Cardiovascular Surgery Heart & Diabetes Center, North Rhine‐Westphalia Bad Oeynhausen Germany; ^3^ Klinik für Innere Medizin III (Kardiologie, Angiologie Und Internistische Intensivmedizin), Universitätsklinikum Des Saarlandes Saarland University Homburg Germany; ^4^ Department of Cardiothoracic and Vascular Surgery Deutsches Herzzentrum der Charite Berlin Germany; ^5^ Department of Cardiothoracic, Transplantation and Vascular Surgery Hannover Medical School Hannover Germany; ^6^ Center for Medical Physics and Biomedical Engineering, Department of Cardiac and Thoracic Aortic Surgery Medical University of Vienna Vienna Austria

**Keywords:** DESTINE staging, driveline infection, left ventricular assist device, prevention, revalidation, standard operating procedure

## Abstract

**Background:**

Driveline infections (DLIs) remain the predominant device‐specific complication in patients supported by left ventricular assist devices (LVADs) serving as a major source of morbidity, recurrent hospitalization, and mortality. Despite their clinical impact, the prevention and early management of DLIs lack standardization, particularly within outpatient and home‐based care environments.

**Methods and Objectives:**

The original 2020 DESTINE proposal introduced a standardized dressing protocol and a prevention‐oriented staging system for driveline exit‐site management. However, subsequent clinical real‐world experience revealed limitations, including overly complex early‐stage subcategorization and the absence of contemporary guidance on wound care, including fixation strategies, antiseptic agents, and adjunctive therapies.

**Updated Recommendations:**

DESTINE 2.0 provides expert recommendations to address these gaps. The updated framework features a streamlined five‐stage system that consolidates previous subcategories into unambiguous, actionable clinical stages. Enhanced recommendations are provided for driveline immobilization, stage‐stratified antiseptic agent selection, and skin barrier preservation. Furthermore, the protocol incorporates adjunctive therapies including cold atmospheric plasma and closed‐incision negative pressure wound therapy. Expanded guidance covers modifiable patient‐level risk factors, perioperative prophylaxis, showering safety, caregiver education, digital documentation, and stage‐directed antibiotic therapy.

**Conclusion:**

By providing a practical, integrated framework applicable to multidisciplinary care settings, DESTINE 2.0 aims to optimize infection‐free survival, minimize healthcare utilization, and improve quality of life for patients on long‐term mechanical circulatory support.

## Graphical abstract

DESTINE 2.0 decision flowchart: stage‐based assessment and management of driveline exit‐site disorders. The flowchart provides a simplified decision‐support tool for the stage‐based evaluation and management of driveline exit‐site disorders in LVAD patients, intended as a practical complement to the staging pyramid (Figre 2) and the detailed staging table (Fiure 3). Starting from visual exit‐site assessment, the flowchart guides clinicians through the five DESTINE 2.0 stages (0–4), with escalating management actions from routine outpatient care at Stage 0 through hospitalization at Stage 3 to surgical intervention and device exchange at Stage 4. The flowchart is intended as a decision‐support aid and does not replace clinical judgment or the full staging definitions provided in Fiures 2 and 3. CAP, cold atmospheric plasma; DLES, driveline exit site; IV, intravenous; NPWT, negative pressure wound therapy; PET‐CT, positron emission tomography‐computed tomography; SOP, standard operating procedure; TEE, transesophageal echocardiography; VAD, ventricular assist device.

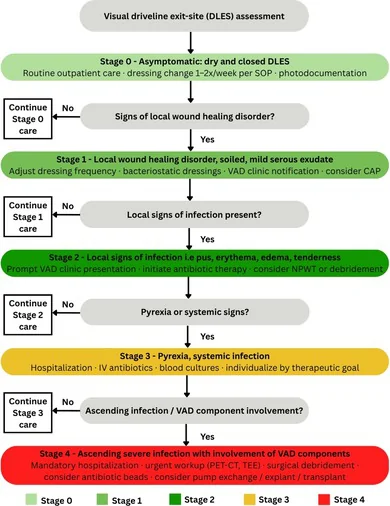

## 1 Background

Advanced heart failure remains a leading cause of morbidity and mortality worldwide, with a growing population of patients progressing to end‐stage disease requiring advanced therapies [1, 2, 3]. Mechanical circulatory support (MCS), particularly continuous‐flow left ventricular assist devices (LVADs), has become an established therapeutic option for patients with refractory heart failure, either as a bridge to transplantation, bridge to candidacy, or destination therapy [4, 5, 6, 7, 8, 9]. Over the past two decades, improvements in pump design, hemocompatibility, and clinical management have significantly enhanced survival and quality of life for LVAD recipients, leading to a steadily increasing population of patients living long‐term with mechanical circulatory support [10, 11]. Despite these advances, device‐related complications continue to limit outcomes. Among them, infections remain one of the most frequent and clinically challenging adverse events [12, 13, 14]. In particular, driveline infections (DLI) represent the predominant LVAD‐specific infection and constitute a major limitation of current LVAD therapy [15, 16, 17, 18]. The percutaneous driveline creates a permanent breach of the skin barrier, predisposing the driveline exit site (DLES) to microbial colonization and mechanical irritation as well as providing a potential starting point for both superficial and deep device infections. Consequently, DLI are associated with substantial morbidity, including recurrent hospitalizations, systemic infection, and, in severe cases, pump exchange or death [19, 20, 21]. Current consensus documents, including those from the International Society for Heart and Lung Transplantation (ISHLT), provide important general recommendations for the prevention and management of MCS‐related infections [22, 23]. However, many aspects of daily clinical practice, particularly in the outpatient setting, remain insufficiently standardized. This applies especially to driveline exit‐site care, including dressing techniques, specific material selection, driveline immobilization, and early recognition of wound healing disorders [24, 25, 26]. As a result, considerable heterogeneity persists across centers and even between caregivers within the same institution, potentially contributing to variable clinical outcomes [15, 27, 28]. To address this gap, the DESTINE (Driveline Expert STagINg and carE) initiative was established in 2020 by an interdisciplinary group of LVAD specialists from Germany and Austria. The original DESTINE proposal introduced a standardized approach to DLES care and proposed a staging system for early detection and stage‐adapted management of driveline infections, with a particular focus on prevention and outpatient care [29]. Since its publication, the DESTINE framework has gained clinical acceptance and contributed to increased awareness of the importance of standardized DLES management [30, 31, 32, 33]. However, real‐world experience has identified limitations in practical applicability. In particular, the complexity of early‐stage subcategorization reduced practical applicability, and advances in wound care, infection prevention strategies, and outpatient management have not been fully reflected. The transition toward a home‐care‐centric model has further intensified the demand for streamlined, standardized, and pragmatically implementable guidelines. To address these deficiencies, the DESTINE expert panel reconvened during the 2026 annual meeting of the German Society for Thoracic and Cardiovascular Surgery to revise and update the original framework. Building upon the experience gained with the original DESTINE proposal, DESTINE 2.0 introduces a simplified and clinically applicable staging concept alongside updated recommendations for driveline fixation, exit‐site care, infection prevention, and stage‐stratified therapeutic treatment. By establishing a practical framework for clinicians, VAD coordinators, and community‐based caregivers involved in the long‐term management of LVAD patients, these updated expert recommendations aim to optimize infection‐free survival, mitigate healthcare utilization, and enhance quality of life for the growing population of patients supported by long‐term MCS. Although convened in the German‐speaking European context, the recommendations are intended for all healthcare professionals, VAD coordinators, nursing staff, and caregivers involved in LVAD driveline management, irrespective of geographic region or healthcare system. In addition, to enhance future research on driveline infections and measurable parameters on different stages of infections, a definition is needed. This accounts for the growing research in this field. The DESTINE 2.0 staging system is therefore designed not only as a practical clinical tool but also as a foundation for systematic outcome research. Driveline infection is currently recorded as a binary variable in major LVAD registries including EUROMACS and INTERMACS, which fails to capture the clinically meaningful spectrum from early colonization to ascending systemic infection. Adoption of the DESTINE 2.0 staging system as a standard registry variable would enable longitudinal trajectory analyses, stage‐specific outcome comparisons, and evidence‐based refinement of management protocols. Prospective studies applying DESTINE 2.0 as a primary classification tool are furthermore warranted to evaluate its reproducibility, interobserver agreement, and prognostic implications across independent LVAD cohorts.

### 1.1 Development of Expert Recommendations and Update Process

The DESTINE 2.0 update represents an expert‐driven refinement of the original framework, integrating accumulated clinical experience with emerging evidence, and structured deliberations from the DESTINE study group. These updated expert recommendations provide a pragmatic synthesis of current literature and real‐world practice across high‐volume VAD centers. By streamlining the staging and therapeutic protocols, DESTINE 2.0 aims to enhance diagnostic precision and procedural standardization across the entire clinical continuum, from acute perioperative in‐hospital management to long‐term community‐based care. This document represents the collective expert opinion of the DESTINE author group, comprising cardiac surgeons and VAD coordinators from six LVAD centers in Germany and Austria, based on accumulated clinical experience and a structured review of current literature. Recommendations were developed through structured discussion at the 2026 annual conference of the German Society for Thoracic and Cardiovascular Surgery, followed by iterative written review and approval by all coauthors. Consistent with the focus on clinical utility, formal grading of the evidence levels was not performed.

### 1.2 Limitations of the DESTINE 1.0 Framework

Clinical feedback and longitudinal application of the original DESTINE 1.0 framework revealed several barriers to its integration into routine practice. A primary concern was the granular subdivision of early‐stage wound conditions (stages 0–2 with a/b subcategorization). While conceptually intended to improve diagnostic precision, this complexity impeded inter‐observer reliability and failed to translate into distinct therapeutic pathways, thereby diminishing its utility in time‐sensitive clinical environments. Similarly, the original Stages 4 and 5, while conceptually distinct, demonstrated substantial overlap in both clinical presentation and therapeutic management, with the differentiation between them proving difficult to apply reliably in practice. Consequently, these categories have been consolidated into a single, actionable Stage 4 in the current iteration. Beyond staging, the visual material and standard operating procedure (SOP) illustrations provided in the original publication no longer reflect current clinical standards. Advances in dressing materials, fixation techniques, and wound care strategies have occurred since the initial release, rendering the original SOP partially outdated. Furthermore, the 2020 framework lacked guidance on emerging preventive and adjunctive modalities such as novel antimicrobial dressings and bio‐active therapies, which have become integral to modern LVAD care. Finally, the initial proposal did not sufficiently account for the practical constraints of home‐based management. In daily (outpatient) practice, driveline care is frequently performed by patients, relatives, or community nursing services, with varying levels of specialized expertise. The original framework did not fully account for these practical constraints, which are central to protocol feasibility and adherence, and therefore fundamental to preventing DLIs in the outpatient setting. The DESTINE 2.0 update systematically bridges these identified gaps to provide a streamlined, evidence‐based standard of DLES care.

### 1.3 Importance of Standardization of Care and Protocol‐Based Management

Consistent, protocol‐based management is a prerequisite for effective DLI prevention. Heterogeneity in dressing techniques, antiseptic application, and immobilization strategies has been identified [24, 26] as a major contributor to inconsistent outcomes across centers and care settings. Consequently, comprehensive SOPs are essential to operationalize all facets of driveline management, including aseptic change techniques, stage‐stratified antiseptic agent selection and application, fixation methods, and systematic inspection of the DLES. To ensure longitudinal efficacy, these protocols should demonstrate pragmatic utility across the entire care continuum—bridging the transition from acute in‐hospital supervision to patient‐ or caregiver‐led DLES management in the home environment. Beyond procedural uniformity, standardized care pathways function as a critical diagnostic safety net, facilitating early recognition of subclinical wound dehiscence or infection while enabling timely intervention.

Several patient‐level risk factors for DLI are modifiable [34] and should be actively addressed both before implantation and throughout the support period. Glycemic control is of particular importance, as poorly controlled diabetes is consistently associated with increased DLI risk [15, 35] and blood glucose levels should be maintained within physiological ranges in accordance with current guidelines [22]. Nutritional status warrants dedicated assessment, with targeted intervention in both cachectic patients and those with significant obesity [36, 37]. Smoking is associated with increased adverse event rates in LVAD patients including DLI [38, 39]. Therefore, cessation should be encouraged prior to implantation. Addressing these factors does not eliminate infection risk but could reduce it, and their systematic management should be considered an integral component of the DESTINE prevention framework. The perioperative phase, broadly defined as the first 30 days following LVAD implantation, represents the critical window for DLES colonization and the establishment of early infection. Preventive efforts during this phase should be optimized through rigorous and consistent driveline immobilization from the time of implantation and structured longitudinal surveillance including standardized photographic documentation at each dressing change to facilitate objective wound monitoring. Adherence to the SOP during this interval, combined with early caregiver training before hospital discharge, establishes the foundation upon which all subsequent outpatient prevention strategies depend.

### 1.4 Standard Operating Procedure and Recommended Dressing Equipment

The dressing change procedure, encompassing infection prophylaxis, caregiver safety, sterile preparation, skin care, wound disinfection, driveline cleansing, dressing application, and secure fixation, reflects best practice consistent with published protocols and represents the recommended standard for VAD centers, outpatient providers, and home caregivers (Figure 1) [15, 24, 27, 29]. Evidence supporting the importance of a standardized, sterile approach to dressing changes has continued to accumulate. Notably, the recently published ASPIRE study demonstrated that structured dressing protocols using a chlorhexidine cleanser with silver‐impregnated dressing were associated with significantly reduced early and late DLI rates, while frequent dressing changes were associated with higher late infection rates [26]. The DESTINE group therefore reiterates that disciplined adherence to a standardized sterile procedure is among the most impactful preventive measures available. While the procedural steps reflect best practice supported by published evidence, the selection of specific dressing materials and products listed in Table 1 represents expert opinion based on accumulated clinical experience across the participating centers, as robust comparative clinical evidence for individual product categories in the LVAD‐specific setting remains limited.

**FIGURE 1:**
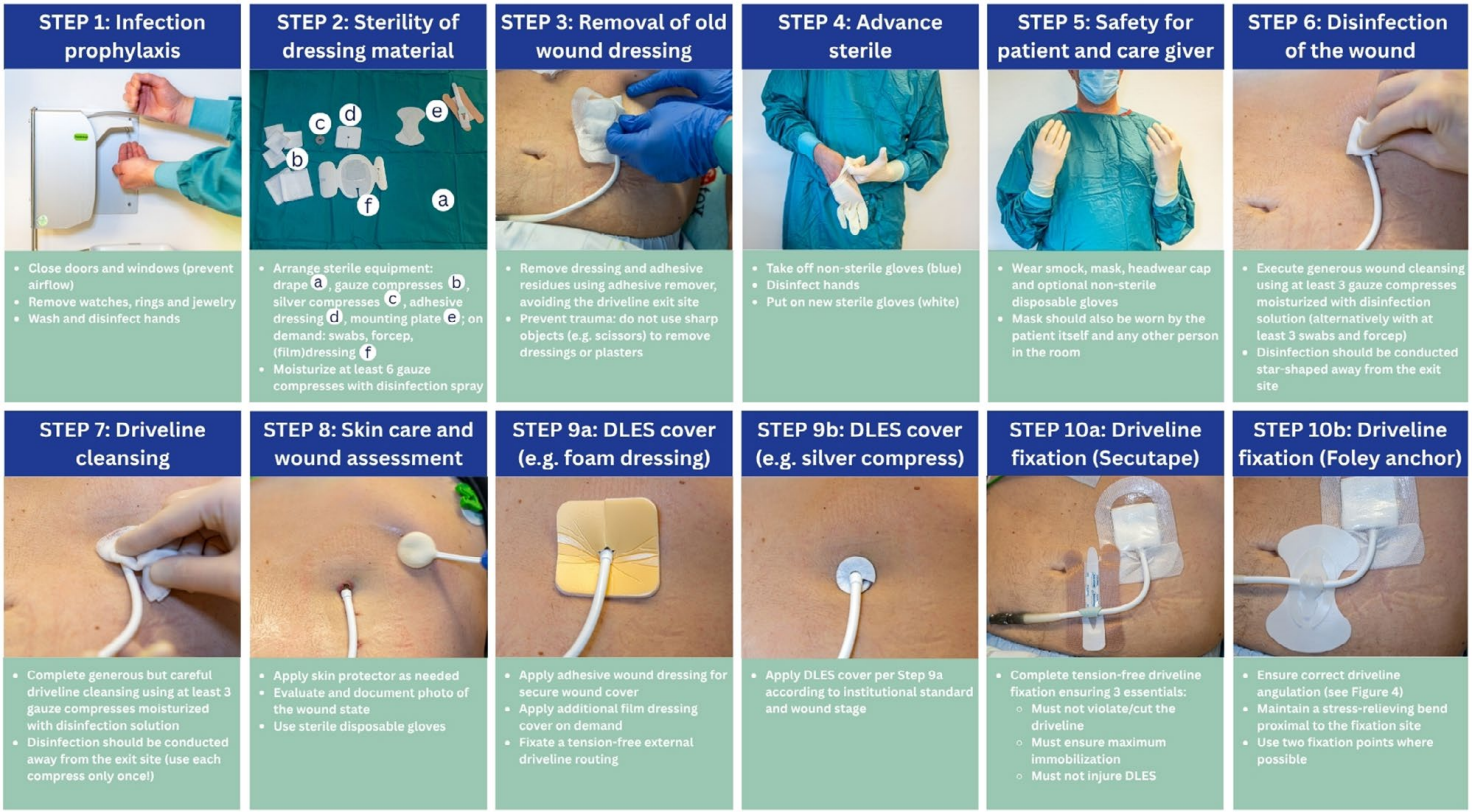
DESTINE 2.0 updated standard operating procedure (SOP) for driveline exit‐site dressing change. The figure illustrates the updated ten‐step DESTINE 2.0 SOP for standardized driveline exit‐site (DLES) dressing change, applicable for VAD clinicians, outpatient nursing services, and caregivers in the home setting. Each step is supported by illustrative clinical photography. The procedure encompasses infection prophylaxis and personal protective equipment (Steps 1–2), sterile preparation of dressing materials (Step 3), removal of the old dressing with skin care and wound assessment (Step 4), hand hygiene and sterile gloving (Steps 5–6), wound and driveline disinfection (Steps 7–8), DLES coverage with stage‐adapted dressing materials, either foam dressing or silver compresses according to wound stage and institutional standard (Steps 9a–9b, representing alternative options), and secure, tension‐free driveline fixation with stress‐relieving bend using either a standard mounting plate or Foley Anchor (Steps 10a and 10b, representing alternative fixation approaches). Material selection should follow institutional protocols and be guided by DESTINE wound stage.

**TABLE 1:** Recommended equipment for standardized driveline exit‐site (DLES) dressing change.

Equipment	Product and Manufacturer (examples)
**Personal protective equipment**	
Smock (disposable, non‐sterile) when alternating patient contact of the caregiver	BeeSana PP/PE‐Kittel, Meditrade GmbH; Smock non‐sterile, CODAN pvb Medical GmbH
Sterile gloves	Gammex PF, Ansell; Protexis PI Classic, Cardinal Health; PEHA‐TAFT, Hartmann AG
Medical disposable gloves	Vasco Vinyl Sensitive, B. Braun; Blue Nitrile Examination Glowes—Powder Free, Mozart, Medovis Healthcare GmbH
Mask (for both patient and caregiver)	Barrier Medical face mask, Mölnlycke Health Care AB; Facial mask with elastic band, Dr. Junghans Medical GmbH
Headwear cap	Barrier Headwear basic, Mölnlycke Health Care AB; Bouffant Cap, green, P. J. Dahlhausen & Co. GmbH
**Draping and preparation**	
Sterile, waterproof overlay/drape, 75 × 90 cm	Mediset Wound treatment set, Hartmann; NOBAPEG, NOBAMED Paul Danz AG
Sterile drape (on demand)	Raucodrape, Lohmann & Rauscher; Foliodrape, Hartmann AG
Adhesive remover spray (sterile, non‐irritating silicone‐based)	For example Esenta Adhesive Remover
Surface disinfectant (liquid and/or wipes)	Mikrozid Universal P Wipes Premium, Schülke & Mayer; Mikrozid AF Liquid, Schülke & Mayer
**Hand and wound disinfection**	
Hand disinfection (wash hands first, then disinfect)	Skinman Soft, Ecolab; Bode Sterillium, Bode Chemie GmbH; SPITACID, Ecolab Deutschland GmbH
Wound disinfection	Octenisept, Schülke & Mayr GmbH, SeraSept 0.04%/SeraSept 0.02% Serag Wiessner
Wound rinsing solution	Octenilin Wound Irrigation Solution, Schülke & Mayr GmbH Prontosan Wound Irrigation Solution, B Braun ActiMaris Sensitiv Wound Irrigation Solution, ActiMaris AG Lavannox Serag Wound Irrigation Solution, Serag Wiesner KerraSol, Solventum
Wound gel	Octenilin Wound Gel, Schülke & Mayr GmbH Prontosan Wound Gel, B Braun ActiMaris Wound Gel, ActiMaris AG Lavannox Serag Wound Gel, Serag Wiesner
**Swabs and compresses**	
Sterile swabs (at least three pcs., on demand)	Mediset Wound treatment set, Hartmann AG; NOBAPEG, NOBAMED Paul Danz AG
Sterile wound gauze compresses (min. 10 pcs., 7.5 × 7.5 10 × 10 cm)	Mediset Wound treatment set, Hartmann AG; NOBAPEG, NOBAMED Paul Danz AG; Vliwasoft, Lohmann & Rauscher; Gauze Swabs, Fuhrmann GmbH
**Sterile bacteriostatic wound dressings (silver‐ and polyhexanide‐based, on demand)**	
Prophylactic/Therapeutic	Hydrofiber dressings: AQUACEL Extra, AQUACEL Ag Extra, Convatec; Fibrosol Silver‐coated sterile antimicrobial barrier dressings: Silverlon IV CD 70, Silverlon Flex, Lifesaver Ag, Bravida Medical Aluminum‐coated dressings: Metalline, Lohmann & Rauscher PHMB‐containing dressings: SUPRASORB X + PHMB HydroBalance Wound Dressing, Lohmann & Rauscher
Exudate management	Calcium alginate (fistula, bloody exudate—protect wound edges): e.g., Kaltostat, ConvaTec; (Silver) e.g., Silvercel, 3 M/Systagenix; Biatain Alginate AG, Coloplast GmbH Activated charcoal wound dressing (for wound odor): e.g., Askina Carbosorb, CarboFlex, Cutimed Sorbion Carbon+ Hydrophobic wound contact layer with DACC coating: Cutimed Sorbact Ribbon, Cutimed Sorbact Contact, Cutimed Sorbact Pad Superabsorbent dressing: Vliwasorb, Cutimed Sorbion Sachet S, RespoSorb Super Polyurethane foam dressing, adhesive: Mepilex Border Flex, Mepilex Border AG, Mepilex Transfer AG, Mölnlycke Health Care AB Polyurethane foam dressing, non‐adhesive: Aquacel Foam NADH, Aquacel Foam AG NADH, Convatec; Biatain, Biatain AG, Coloplast
Post‐NPWT: wound healing optimization	Hyaluronic acid with iodine complex: Hyiodine Gel, Contipro a.s. Collagen matrix with silver: PromogranPrisma, 3 M Acellular fish skin (rich in polyunsaturated omega‐3 fatty acids): Kerecis, Coloplast
**Wound coverage and driveline fixation**	
Film dressing—no exudate	3 M Tegaderm I.V. Fixation Dressing, 8.5 × 10.5 cm; SorbaView SHIELD 9.53 × 11.75 cm, Medline; Opsite Film 10 × 12 cm, Smith & Nephew
Sterile adhesive wound dressings—exudation phase	Cosmopor, Hartmann AG; Cutiplast steril, Smith & Nephew; Curapor, Lohmann & Rauscher
Shower patches (secondary dressing)	Suprasorb F, Lohmann & Rauscher; OPSITE IV 3000, Smith & Nephew; Xtrata mediset
Cover plaster (width 15 cm, on demand)	Fixomull Stretch, BSN Medical; Medipore Hypoallergenic Cloth Tape, 3 M Deutschland GmbH
Mounting plate/driveline fixation	Foley Anchor, Centurion; Secutape, TechniMed AG; Horizontal Tube Attachment Device (HTAD), Hollister; CathGrip, BioDerm
**Tools, skin care, and hygiene accessories**	
Sterile forceps (on demand)	Mediset Wound treatment set, Hartmann AG; Single‐use forceps, sterile, Dr. Junghans Medical GmbH; meco Wound Care Set, meco Store
Disposable forceps	Disposable Forceps, Mediware; Dissecting Forceps Adson 12 cm, ClinaStar
Skin protector	Cavilon, 3 M; Secura, Smith & Nephew; NO‐STING SKIN‐PREP, Smith & Nephew
Single‐use skin shaver (on demand)	Med‐Comfort, AMPri GmbH; Disposable Double‐Cutting Razor, pfm medical AG
Wash lotion	Decontaman liquid, Dr. Schumacher GmbH; Octenisan Wash Lotion, Schülke & Mayr GmbH
Shower filter (protection against waterborne pathogens)	i3 TWO Filter, i3 Membrane GmbH

*Note:* Products listed are examples; equivalent products may be used per institutional or regional protocol. Selection of dressing materials should be guided by wound stage (see Table 2) and individual patient requirements. Products and manufacturers are listed as illustrative examples only. The inclusion of trade names is intended to provide practical orientation for clinicians and does not constitute endorsement of any specific manufacturer. Multiple competing products within each category are listed to avoid commercial bias. Equivalent alternatives may be substituted per institutional protocol and local availability.

Abbreviations: DACC, dialkylcarbamoyl chloride; DLES, driveline exit site; NPWT, negative pressure wound therapy; PHMB, polyhexamethylene biguanide (polyhexanide).

Material selection for dressing changes remains center‐specific and should reflect local availability, institutional protocols, and individual patient needs. Recommended dressing categories include film dressings, hydro fiber and foam dressings, bacteriostatic silver‐ and polyhexamethylene biguanide (PHMB)‐containing wound dressings, adhesive wound dressings, and driveline fixation devices. The selection of specific products within each category should be guided by the patient's current DESTINE stage, wound characteristics, and skin tolerance. An updated overview of recommended equipment and example products for standardized DLES dressing change is provided in Table 1. It should be emphasized that the illustrated steps represent a framework rather than a prescriptive protocol; specific materials and procedural details will inevitably vary according to institutional standards, regional availability, and individual patient requirements. Given the breadth of available options, Figure 1 focuses on the essential steps and key principles, with material selection guided by the recommendations provided in Table 1. In patients with known contact dermatitis or adhesive intolerance, alternative wound cleansing solutions and dressing materials with a favorable skin compatibility profile should be selected; a range of suitable alternatives is provided in Table 1 to accommodate individual sensitivities and local availability.

### 1.5 Updated DESTINE 2.0 Staging System

The revised staging system introduces five stages (0–4) but consolidates the a/b subcategories of the original stages 0–2 into single, unambiguous stages. The original Stages 4 and 5 have been merged into a single Stage 4 reflecting ascending severe infection with potential involvement of VAD components [33]. Stage 0 defines the asymptomatic, clinically unremarkable exit site. Stage 1 denotes a local wound healing disorder characterized by soiling and mild serous exudate without systemic signs of infection. Stage 2 represents established local infection with erythema, edema, tenderness, and purulent discharge, still without systemic involvement. Stage 3 is characterized by pyrexia and systemic infection without pump housing involvement, necessitating hospitalization. Stage 4 represents the most severe form, with ascending infection and potential involvement of VAD components including the pump, urgent diagnostic workup, and surgical intervention, including Negative Pressure Wound Therapy (NPWT), debridement, and source control with pump exchange, explant, or transplantation as the definitive therapeutic options. Management intensity escalates across these stages from routine standardized care through adjunctive local therapies to antibiotic treatment and surgical consideration. The complete stage definitions, diagnostic criteria, and recommended actions are presented in Figures 2 and 3.

**FIGURE 2:**
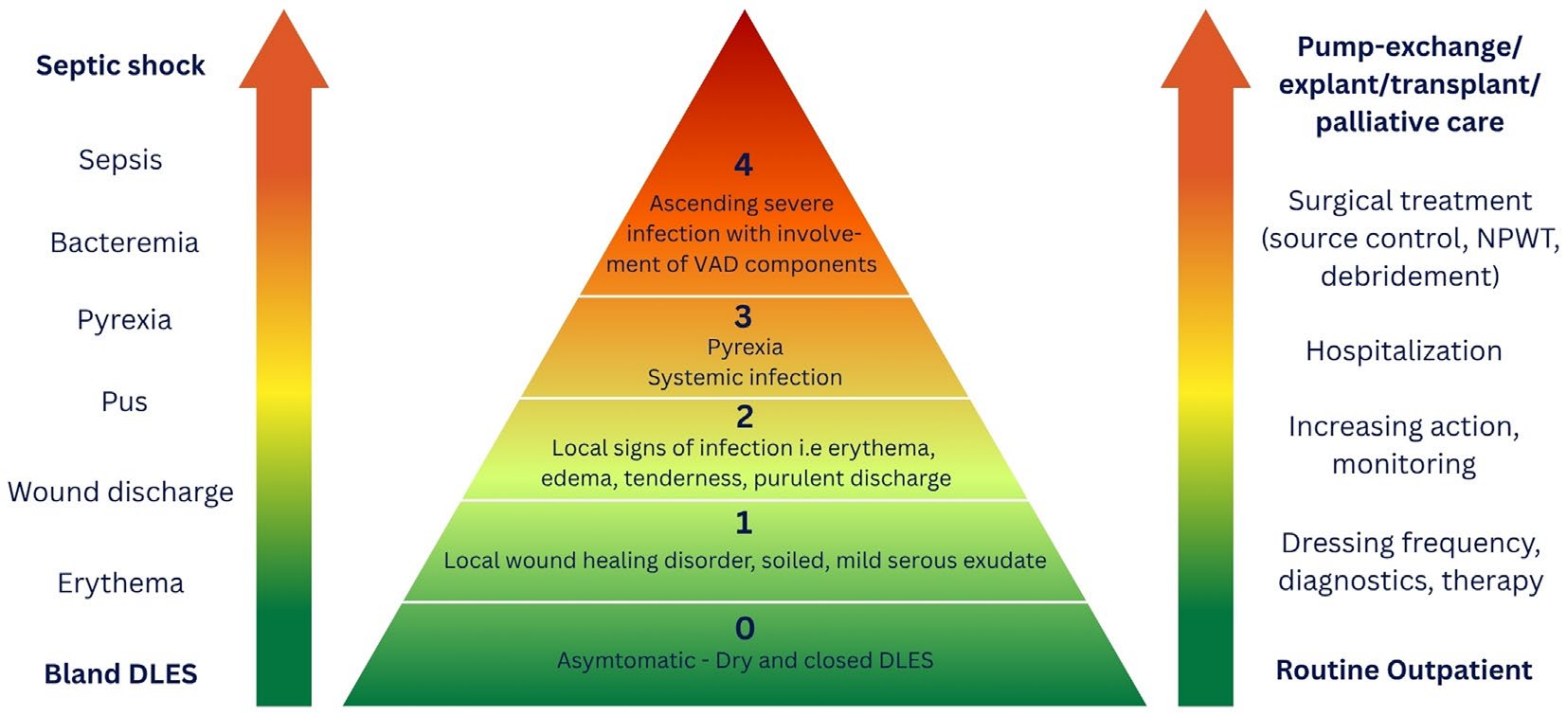
DESTINE 2.0 staging—wound healing disorders and required actions at the driveline exit site. The pyramid schematic illustrates that the majority of LVAD outpatients present with a bland, dry driveline exit site (Stage 0) and therefore require consistent standard‐of‐care management as the foundation of infection prevention. With the onset and progression of a wound healing disorder, increasing clinical action and awareness are mandatory to prevent advancement to local infection, systemic involvement, hospitalization, sepsis, and ultimately death. The left axis depicts the escalating clinical signs associated with advancing stage; the right axis reflects the corresponding increase in required intervention intensity, ranging from routine outpatient care at Stage 0 through hospitalization at Stage 3 to surgical intervention and pump exchange, explant, or transplantation at Stage 4.

**FIGURE 3:**
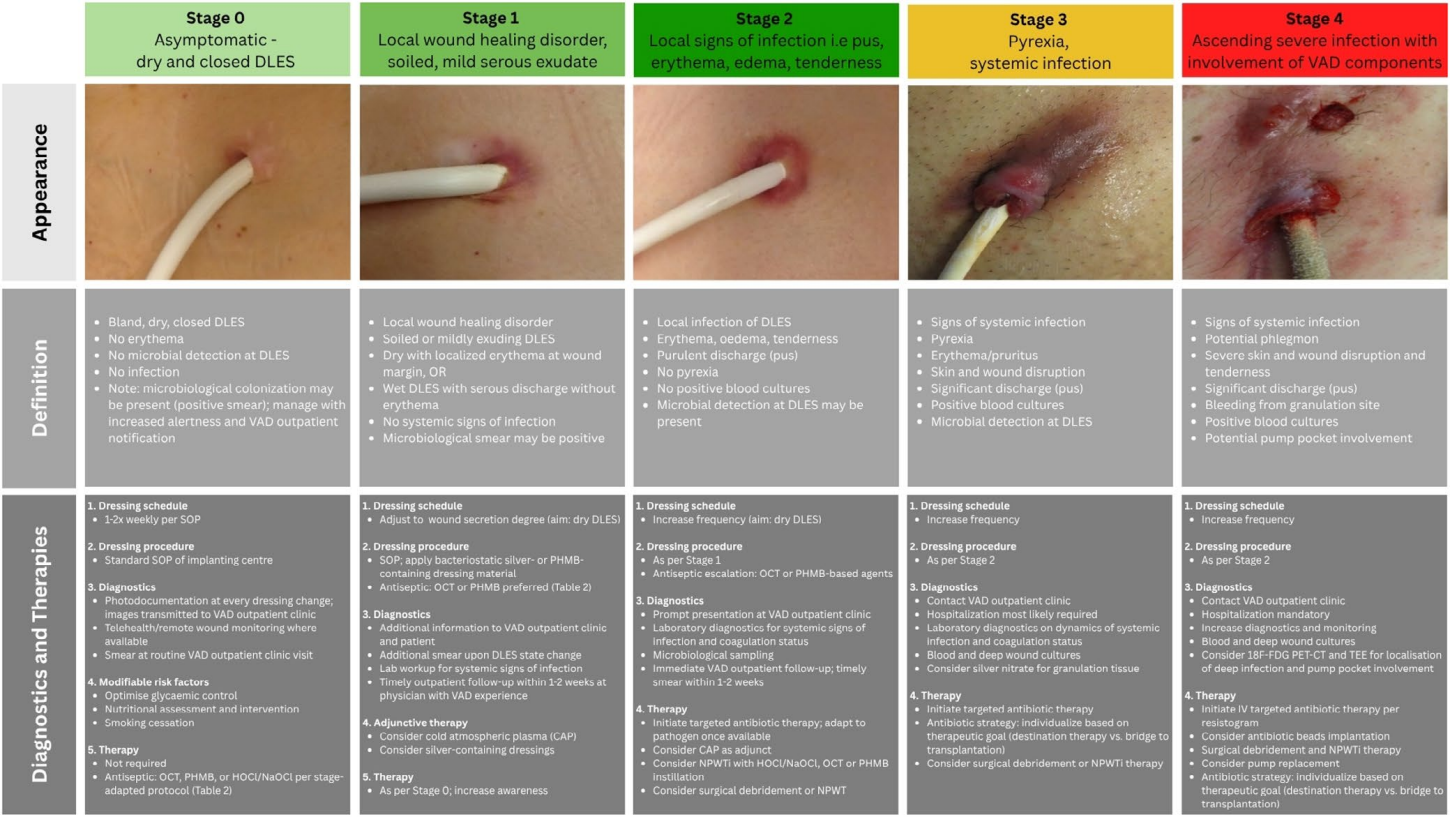
DESTINE 2.0 staging system: clinical definitions, diagnostics, and stage‐adapted management of driveline exit‐site disorders. Each column represents one DESTINE 2.0 stage (0–4), illustrated by representative clinical photography and defined by phenotype‐based criteria. The lower panel details recommended diagnostics and therapies at each stage, escalating in intensity from routine outpatient care at Stage 0 to urgent hospitalization, surgical intervention, and device exchange at Stage 4. CAP, cold atmospheric plasma; DLES, driveline exit site; DLI, driveline infection; HOCl/NaOCl, hypochlorous acid/sodium hypochlorite; NPWTi, negative pressure wound therapy with instillation; OCT, octenidine; PET‐CT, positron emission tomography–computed tomography; PHMB, polyhexanide; SOP, standard operating procedure; TEE, transesophageal echocardiography. See Table 2 for antiseptic agent selection by stage.

Of note, the DESTINE 2.0 staging system was developed as a practical clinical tool and is not intended to replace existing international infection definitions. In relation to the 2024 ISHLT infection definitions for MCS devices [23], which classify percutaneous lead infections as either uncomplicated or complicated, the DESTINE stages can be broadly mapped as follows: Stage 2, characterized by local infection without systemic involvement and with negative blood cultures, corresponds to an uncomplicated percutaneous lead infection; Stages 3 and 4, involving systemic signs and, in the case of Stage 4, potential VAD component involvement, correspond to complicated percutaneous lead infection, with Stage 4 additionally fulfilling criteria for infection of the external surfaces of an implantable component. Stages 0 and 1 fall outside the ISHLT infection threshold entirely, as they address wound healing disorders and preinfection states that are not captured by binary infection classifications but are central to the preventive focus of the DESTINE framework.

### 1.6 Driveline Integrity, Fixation, and Repair

As the only permanent percutaneous component of contemporary LVAD systems, the driveline is exposed to continuous tensile, torsional, and environmental stress over prolonged support durations. The resulting cumulative mechanical burden contributes to repetitive microtrauma at the DLES, disrupting the skin‐device interface and facilitating bacterial entry. Preservation of driveline integrity and minimization of mechanical stress are therefore fundamental components of DLI prevention, extending beyond local wound care alone.

#### 1.6.1 Material Properties and Structural Considerations

Discussion of driveline material properties is currently centered on the HeartMate 3, which represents the only widely available durable LVAD system in contemporary practice. The HeartMate 3 driveline is constructed as a multilayer system comprising internal electrical leads, a tension‐relieving core, polyurethane insulation, Kevlar reinforcement, and an outer silicone sheath [34]. This design provides a high degree of durability and resistance to external mechanical stress while enabling long‐term use under ambulatory conditions. At the same time, the mechanical characteristics of drivelines represent modifiable factors that may influence DLI risk [40]. Repetitive mechanical loading, including bending and torsion during daily activities, can alter the mechanical behavior of the driveline over time [34], potentially affecting force transmission at the DLES and the *tissue* compatibility at the exit interface [40]. In addition, the outer silicone layer, while facilitating cleaning and handling, may be susceptible to surface wear or minor damage during routine use. Such alterations do not represent device failure per se but may contribute to local vulnerability if not identified and managed appropriately.

#### 1.6.2 Positioning of the Velour Interface and Driveline Exit Site

The position of the driveline at the skin interface is a critical but frequently underrecognized factor in infection prevention. Particular attention should be paid to the transition between the intracorporeal velour segment and the external driveline. The velour‐covered portion is designed to promote tissue integration and is recommended to remain at or just below skin level. Exposure of the velour to the external environment leads to irreversible contamination, creating a persistent substrate for bacterial colonization that cannot be effectively decontaminated. Accordingly, correct positioning of the velour interface at the time of implantation and during follow‐up is essential and may be routinely assessed as part of driveline exit‐site evaluation [41]. With regard to the optimal exit‐site location, a flat, accessible surface, generally the left abdominal wall, lateral to the midline and between the umbilicus and the costal margin, can facilitate effective wound cleansing, dressing application, and driveline fixation [41]. Proximity to the umbilicus or midline should be avoided. Exit‐site selection should be discussed with the patient preoperatively, accounting for body habitus and clothing preferences.

#### 1.6.3 Driveline Immobilization and Fixation Strategies

Effective immobilization of the driveline is essential for preventing driveline infections, as it mitigates both acute strain (e.g., accidental traction) and cumulative low‐intensity forces during daily activities that can lead to chronic irritation and impaired wound stability. Driveline fixation devices significantly reduce tensile forces compared with no fixation [42]. Experimental data suggest a clustering of devices into those providing higher versus lower levels of mechanical protection, with systems such as CathGrip, Secutape, and Hollister demonstrating lower force transmission, and thus preferable characteristics, compared with other commonly used devices [42]. Selection of fixation devices should consider mechanical stability, form factor, adhesive properties and skin compatibility, as well as the reliability and safety of cable securing. Notably, any fixation device is preferable to none, as all tested systems provide substantial tensile load reduction. When hair removal around the DLES is required, shaving should be performed with extreme caution to avoid inadvertent nicking of the outer silicone insulation, which would compromise driveline integrity and create a potential entry point for infection. In patients with sensitive skin or adhesive intolerance, placing the fixation device on a protective layer of full‐surface tape can reduce skin irritation while maintaining adequate fixation. Positioning is equally critical (Figure 4). Based on published experimental force transmission data, a perpendicular (approximately 90°) alignment of the driveline relative to the exit site markedly reduces force transmission compared with a straight configuration [42]. Effective immobilization may therefore require alignment of the external driveline with the internal tunneled path, followed by guidance at approximately 90° beyond the dressing. The exact direction beyond the dressing is recommended to be adapted to the individual patient, accounting for exit‐site location, body habitus, and clothing preferences. The fixation device should be placed as close as possible to the wound dressing, generally within approximately 10 cm of the exit site, to minimize the length of unsupported driveline and reduce force transmission to the exit site. This configuration allows the driveline to function as a mechanical buffer, preventing direct transmission of pulling forces to the DLES. Where anatomy permits, incorporation of a stress‐relieving bend proximal to the fixation point could further enhance this effect. Figure 4B illustrates the principle of force redirection at 90°, which represents one clinically applicable approach. A straight‐line alignment, by contrast, transfers movement directly to the wound, an effect further amplified by skin mobility, particularly in patients with increased subcutaneous tissue. Importantly, even fixation systems with comparatively lower mechanical performance may provide adequate protection when correctly applied [42], particularly if sufficient redundant driveline length is maintained to enable effective decoupling of movement. It should be noted that the exact configuration of the stress‐relieving bend will vary between patients depending on anatomy and body habitus and the principle of force redirection is more important than the precise geometry of the bend, and multiple configurations may provide comparable mechanical protection. Conversely, suboptimal positioning may negate the benefit of otherwise superior devices. Future studies should aim to correlate mechanical performance of driveline fixation devices with clinical DLI outcomes to establish evidence‐based recommendations. It is worth noting that the surgical implantation technique itself may facilitate optimal external driveline positioning and reduce mechanical stress at the exit site from the outset. The 2023 ISHLT Guidelines for Mechanical Circulatory Support recommend that the entire velour portion of the driveline should remain subcutaneous, resulting in a silicone‐skin interface at the exit site, and that the driveline should be anchored close to the exit site to mitigate trauma [41]. Extended tunneling through the rectus sheath, the so‐called double‐tunnel technique, placing the driveline within the sheath of the rectus muscle in the umbilical direction and then subcutaneously to the upper quadrant, has been reported by several centers with low driveline infection incidence, including a demonstrated reduction in 1‐year infection rates from 22.5% to 4.9% [43] and sustained benefit at 5‐year follow‐up [44], and is classified as a Class IIb recommendation in the 2023 ISHLT Guidelines [41, 43, 44, 45]. Where feasible and consistent with institutional practice, tension‐free driveline routing using such techniques may naturally facilitate the external driveline angulation and mechanical decoupling described in this document. However, a detailed discussion of intraoperative technique falls outside the scope of this expert opinion document, which addresses exclusively postimplantation driveline management. Centers are referred to the 2023 ISHLT Guidelines for Mechanical Circulatory Support for detailed surgical guidance [41].

**FIGURE 4:**
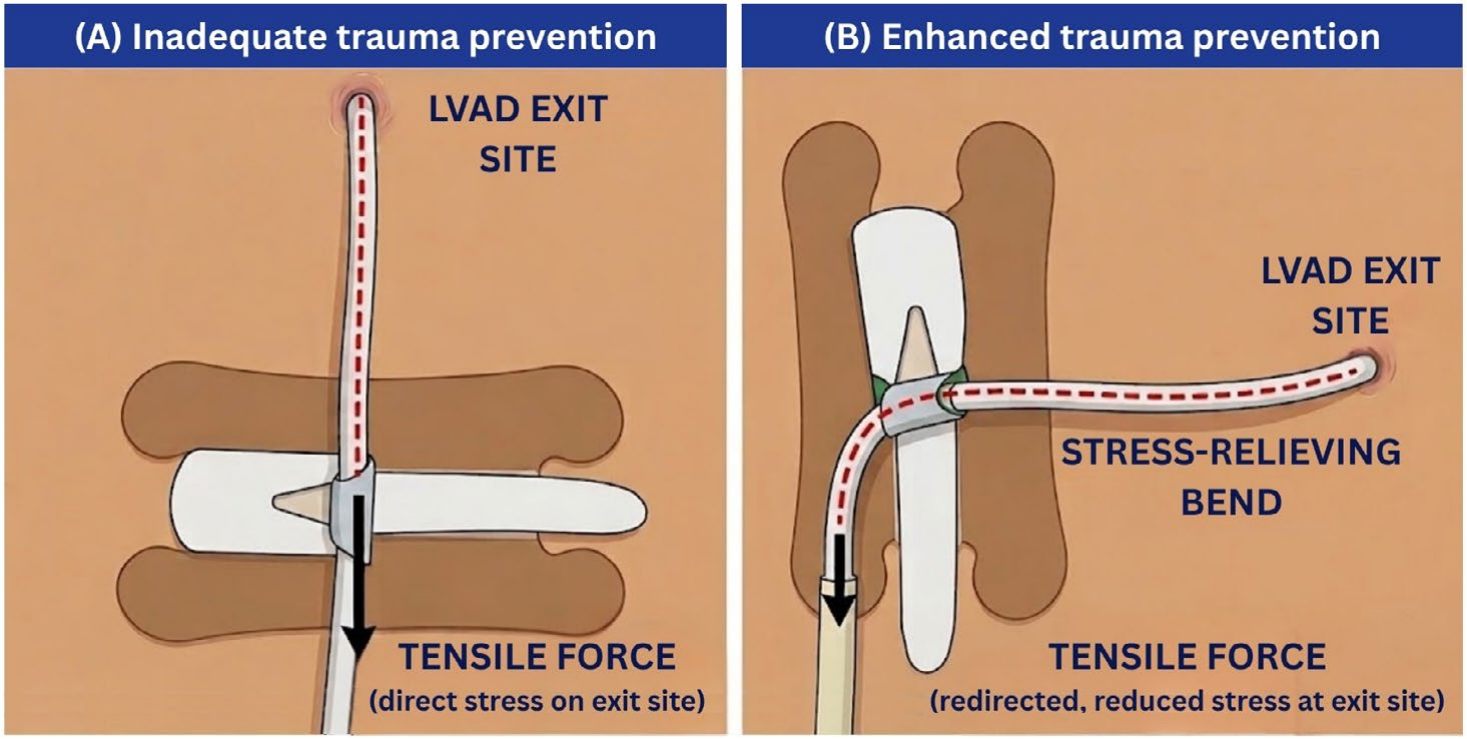
Driveline fixation and positioning: inadequate versus enhanced trauma prevention at the driveline exit site. Panel (A) illustrates inadequate driveline configuration in which straight‐line alignment results in direct transmission of tensile forces to the driveline exit site, predisposing to repetitive microtrauma and wound instability. Panel (B) demonstrates enhanced fixation with approximately 90° driveline angulation beyond the anchoring device, redirecting and dissipating tensile forces away from the exit site. This configuration is presented as an illustrative example; incorporation of a stress‐relieving bend proximal to the fixation point (where anatomically feasible) further reduces force transmission and represents the preferred approach. Selection of fixation strategy should be individualized based on patient anatomy, body habitus, and device‐specific characteristics.

#### 1.6.4 Repair Strategies and Long‐Term Durability

With support durations now regularly extending beyond 5 years, as evidenced by 5‐year survival rates of approximately 58% and 63% in pivotal trial and real‐world registry settings respectively [8, 46], rising to approximately 78% in younger patient cohorts [47], driveline degradation, particularly of the outer insulation, has become a foreseeable clinical reality. Proactive identification and appropriate repair are essential to maintain structural integrity and mitigate infection risk. Superficial damage of the silicone insulation, including cuts or material thinning, should be managed with silicone‐based rescue tape to establish a flexible, hydrophobic seal. Standard adhesive tapes are contraindicated due to poor long‐term adhesion and material rigidity. Even in cases of advanced wear or partial disintegration of the outer insulation, reconstruction with silicone‐based rescue tape remains the preferred approach to restore surface integrity and limit contamination and microbial colonization. Defects proximal to the DLES necessitate specialized management to avoid additional tissue trauma. Fast‐curing two‐component silicone adhesives are therefore recommended, while alternatively elastic silicone or latex tubing can be used to cover the damaged segment, providing protection without additional mechanical stress [48]. Similarly, degradation of strain‐relief components at the connector interface, often accelerated by chemical exposure to disinfectants and skin products, should be addressed with silicone sealants to prevent the formation of contaminant reservoirs. Structural damage, involving Kevlar reinforcement fibers or internal leads (typically signaled by device alarms), warrants urgent consultation with the manufacturer. Such interventions are generally feasible only if the damaged segment is located at a sufficient distance from the DLES (approximately ≥ 15 cm). All repairs should utilize manufacturer‐approved biocompatible materials to ensure device safety and preserve warranty status, prioritizing a pragmatic approach that minimizes localized trauma.

### 1.7 Skin Protection and Preservation of Barrier Function

In addition to microbial colonization, repetitive mechanical and chemical stress contributes substantially to local skin damage. Frequent dressing changes [26], adhesive‐related trauma, and prolonged exposure to antiseptic agents may cumulatively impair the epidermal barrier, which could result in dermatitis, maceration, and erosion, alterations that increase susceptibility to DLI and may compromise patient adherence and quality of life. Preventive strategies should therefore balance antimicrobial efficacy with tissue compatibility, and dressing approaches should be individualized to minimize cumulative skin injury. The selection of antiseptic agents should be guided by three key criteria: compatibility with driveline components to prevent structural degradation; effective microbial reduction within a short contact time suitable for repeated long‐term application; and broad activity across the exit site, the surrounding peri‐wound skin, and the exposed driveline segment beneath the dressing.

#### 1.7.1 Hypochlorous Acid–Based Wound Cleansing Solutions (HOCl/NaOCl)

Hypochlorous acid (HOCl)‐based wound cleansing solutions represent a suitable option for routine DLES care, particularly due to their favorable biocompatibility profile and suitability for repeated long‐term application. As hypotonic solutions, they are well tolerated and may additionally contribute to odor reduction in colonized or infected wounds. Antimicrobial efficacy, however, varies substantially between products and is primarily driven by concentration, total chlorine content, and exposure time. Higher concentrations (e.g., ~0.08%) are associated with improved antimicrobial activity. However, increasing efficacy is linked to increased cytotoxic effects, particularly toward fibroblasts, highlighting the need for careful product selection [49]. In vitro data demonstrate a clear concentration‐ and time‐dependent relationship, with higher antimicrobial activity accompanied by reduced cell viability [49]. Importantly, not all commercially available HOCl‐based solutions demonstrate sufficient antimicrobial efficacy despite excellent biocompatibility. Short exposure times (≤ 5 min) appear to offer a favorable balance between antimicrobial effect and tissue tolerance in clinically relevant settings. Hypertonic wound irrigation solutions (up to ~3% NaCl) exert osmotic, decongestive, and antimicrobial effects but carry a risk of tissue stress that limits their use to short‐term application in infected or heavily contaminated wounds. Evidence supporting routine use at DLES remains limited. Overall, HOCl‐based solutions are recommended to be selected based on their specific efficacy profile rather than assumed class effects.

#### 1.7.2 Octenidine‐ and Polyhexanide‐Based Antiseptic Solutions

Octenidine‐ and PHMB‐based antiseptics represent key alternatives to hypochlorite‐based solutions in contemporary wound care and are currently considered first‐line options in critically colonized or infected DLES [50]. Chlorhexidine gluconate (CHG), although widely used in clinical practice for percutaneous device care, is not included among the recommended agents [26]. Concerns regarding potential degradation of silicone driveline components, combined with a less favorable cytotoxicity profile relative to octenidine and PHMB, limit its applicability in the DLES setting centers currently using CHG‐based protocols are encouraged to review compatibility with their specific device (driveline/material). Earlier formulations containing octenidine in combination with phenoxyethanol (e.g., Octenisept) are now used more cautiously due to potential material incompatibility and should be applied in a time‐limited manner, avoiding occlusive coverage after application. Polyhexanide is characterized by broad antimicrobial activity, including efficacy against *Staphylococcus aureus* and *Escherichia coli* , and demonstrates favorable biocompatibility with low cytotoxicity [51]. In addition, PHMB exhibits relevant anti‐biofilm activity and may form a persistent antimicrobial film, particularly when used in gel‐based formulations. Its clinical use is, however, limited by comparatively long required exposure times. Octenidine‐based solutions demonstrate rapid antimicrobial activity and have shown consistent efficacy even under conditions of organic contamination [52]. Standardized in vitro comparisons indicate that octenidine‐containing solutions achieve sufficient antimicrobial activity within short exposure times (≤ 30 s), whereas hypochlorite‐based solutions may lose efficacy in the presence of organic [53]. Similar findings have been reported in studies evaluating wound exudate conditions, as well as in biofilm models, where both octenidine‐ and PHMB‐based antiseptics demonstrated superior performance compared with low‐concentration hypochlorite solutions [54]. These observations are supported by experimental [55] and clinical [56] data, including studies demonstrating rapid antimicrobial efficacy of octenidine in combination with negative pressure wound therapy with instillation (NPWTi), as well as consistent anti‐biofilm activity of both octenidine and PHMB. Hypochlorite‐based solutions appear less suitable where targeted anti‐biofilm activity is required [55]. Overall, octenidine‐ and PHMB‐based antiseptics provide effective options for targeted antimicrobial management in driveline care. Octenidine may be preferred when rapid antimicrobial action is required, particularly in the presence of organic contamination, whereas PHMB offers advantages in terms of biocompatibility and anti‐biofilm activity. Exposure times differ substantially between agents, that is approximately 1 min for octenidine, PVP‐I, and HOCl versus up to 15 min for PHMB, alongside relevant differences in biofilm activity, remanent effects, and tissue compatibility. Emerging evidence suggests that bacterial response to antiseptics is species‐ and strain‐dependent, with potential for adaptive changes under repeated exposure, further supporting the need for a differentiated and context‐specific selection of antiseptic agents [57]. The key pharmacological properties, recommended exposure times, and stage‐adapted clinical applicability of the antiseptic agents discussed above are summarized in Table 2. Of particular practical relevance are the agent‐specific contact times listed in Table 2, which vary substantially between agents and should guide antiseptic selection in the home‐based and outpatient setting where prolonged application times may limit compliance. The recommendations presented in this section are grounded in published in vitro and observational evidence where available; in areas where robust comparative clinical data are lacking, recommendations reflect the accumulated expert opinion of the author group.

**TABLE 2:** Wound irrigation solutions and antiseptics for driveline exit‐site management in VAD patients stratified by DESTINE stage.

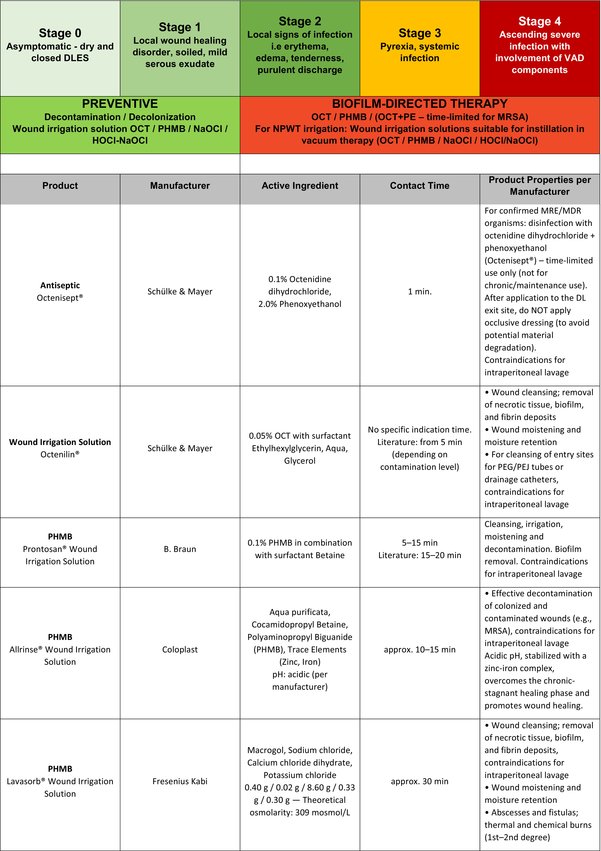
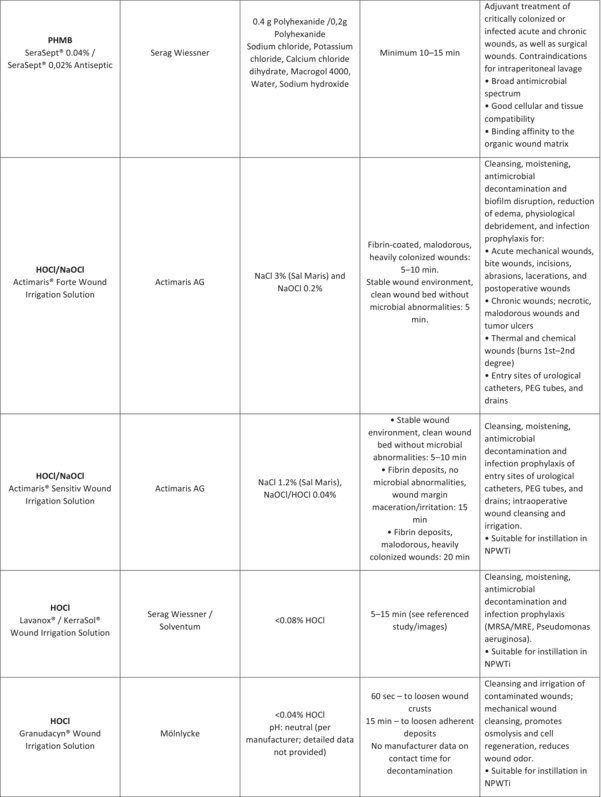

*Note:* Products and manufacturers listed are examples only. Antiseptic formulations are presented by active substance class; clinically equivalent alternative formulations within the same substance class may be used, provided that concentration, contact time, and contraindication profile are comparable. Product properties listed are per manufacturer specifications and should be verified against current product documentation.

Abbreviations: DLES, Driveline exit site; MRE/MDR, Multi‐drug‐resistant organism; MRSA, Methicillin‐resistant *Staphylococcus aureus* ; NaOCl/HOCl, Sodium hypochlorite/Hypochlorous acid; NPWTi, Negative‐pressure wound therapy instillation; OCT, Octenidine; PE, Phenoxyethanol; PEG/PEJ, Percutaneous endoscopic gastrostomy/jejunostomy; PHMB, Polyhexamethylene biguanide (Polyhexanide).

### 1.8 Closed‐Incision Negative Pressure Therapy (ciNPT)

Closed‐incision negative pressure wound therapy (ciNPT), most commonly applied using the Prevena system, is an adjunctive strategy for preventing surgical site complications in high‐risk patients. The system applies continuous negative pressure (−125 mmHg), enabling effective exudate removal, reduction of wound edema, and stabilization of the incision environment. Beyond fluid evacuation and dead‐space reduction, the sealed environment limits external contamination; antimicrobial silver‐containing dressings integrated into the system may further support local wound healing. Clinical evidence from cardiothoracic surgery consistently demonstrates reduced surgical site infection rates with ciNPT, with reductions of approximately 49% overall and up to 61% in high‐risk patient cohorts reported across multiple studies and meta‐analyses [58, 59, 60, 61, 62]. In the context of driveline management, ciNPT may support early exit‐site stabilization by reducing local fluid accumulation, minimizing mechanical irritation, and creating a controlled wound environment during the immediate postoperative phase. This may be particularly relevant in high‐risk patients such as those with obesity or compromised tissue conditions, where early wound instability is a known contributor to subsequent driveline infection. Direct evidence specific to DLES remains limited and ciNPT may therefore be considered selectively in high‐risk patients as part of a structured perioperative prevention strategy rather than applied as routine practice.

### 1.9 Cold Atmospheric Plasma as an Adjunctive Treatment Approach for Driveline Infections

The management of DLIs increasingly requires adjunctive local strategies that address not only microbial burden but also impaired wound healing and persistent inflammation. In this context, cold atmospheric plasma (CAP) has gained attention as a potential complementary modality due to its combined antimicrobial and tissue‐modulating properties [63, 64, 65]. CAP technologies, particularly argon‐based devices, generate reactive oxygen and nitrogen species, ionized particles, and low‐dose ultraviolet radiation at tissue‐compatible temperatures, exerting broad‐spectrum antimicrobial effects including activity against biofilm‐associated microorganisms and antibiotic‐resistant pathogens [66, 67, 68, 69, 70, 71, 72]. Beyond microbial reduction, CAP appears to modulate local wound biology through effects on microcirculation and regenerative signaling pathways [71, 73, 74, 75, 76], with controlled trials in chronic wounds demonstrating bacterial load reduction comparable to established wound care strategies [64, 68]. Within the LVAD population, initial case series demonstrated feasibility and safety of CAP in superficial DLIs, including extended application in pediatric paracorporeal systems [77, 78]. Emerging observational evidence further suggests that integration of CAP into local wound care protocols may be associated with improved infection‐related outcomes and increased freedom from driveline infection [30], with additional reports indicating potential benefit in recurrent or therapy‐refractory cases where conventional approaches are insufficient [40, 79, 80]. Most recently, a single‐center cohort study [33] of 78 LVAD outpatients with DLI demonstrated that adjuvant CAP therapy resulted in significantly higher wound healing rates at 1 year compared with standard care alone (50% vs. 25%, *p* = 0.03), alongside a marked reduction in DLI‐related hospital readmissions (19.0% vs. 54.5%, *p* < 0.001). Notably, patients with higher DESTINE stages at baseline required more CAP sessions to achieve healing, supporting early initiation of CAP therapy as a clinical priority. No device‐related adverse effects or pump malfunctions were observed across 943 treatment sessions, confirming the safety profile of CAP in this setting [33]. The current evidence base remains limited by heterogeneous study designs and small patient numbers. CAP may therefore be regarded as an adjunctive therapy within a multimodal treatment concept rather than a standalone intervention, with use particularly considered in early‐stage infections, biofilm‐associated conditions, and cases with impaired wound healing.

### 1.10 Showering and Hygiene Management

Showering in LVAD patients requires careful precautions, as external system components are moisture‐resistant but not waterproof. Direct exposure of the controller, driveline, and batteries to water risks device malfunction or electrical hazard and should be strictly avoided; dedicated waterproof protective systems such as shower bags are therefore mandatory when showering is permitted. Showering may only be permitted after complete healing of the DLES and upon explicit approval by the treating VAD team. From an infection prevention perspective, maintaining a dry and stable DLES remains essential. Excessive moisture exposure impairs skin integrity and promotes microbial colonization, and the domestic bathroom environment represents an additional source of contamination, particularly with waterborne pathogens. Single‐center data have linked conventional showering to increased rates of *Pseudomonas aeruginosa* DLIs, with a reduction observed following discontinuation of unprotected showering, and bathroom‐associated contamination has been identified as a relevant transmission pathway [81, 82]. Showering should accordingly be performed only under strict protective measures, with structured patient education forming an essential component of safe long‐term practice.

### 1.11 Training and Education

Structured training and education are central to safe long‐term LVAD care and should be considered a core component of DLI prevention. Education should begin during the index hospitalization and continue through discharge and long‐term follow‐up, with explicit inclusion of caregivers and home‐care providers. This approach is consistent with current position statements and post‐discharge management recommendations emphasizing discharge preparation, caregiver involvement, and continuity of education beyond hospitalization [83, 84, 85]. Given the complexity of daily self‐management in LVAD patients, structured educational curricula should be implemented to support adherence and reduce adverse events [86, 87]. Content should extend beyond device operation to encompass dressing and driveline care, early recognition of infection signs, alarm management, anticoagulation, and medication adherence [88, 89, 90, 91]. Checklist‐based educational tools have demonstrated feasibility, patient acceptability, and improved team communication and engagement [92, 93]. Advances in digital health offer important adjuncts to structured education and outpatient follow‐up. Photo documentation of the DLES, which was already recommended in the original DESTINE framework, has evolved from an optional practice into a standardized component of remote wound monitoring in leading LVAD centers. Structured telehealth follow‐up, combining standardized imaging protocols with virtual nurse‐ or physician‐led assessment, enables early detection of wound healing disorders between scheduled outpatient visits and supports timely escalation without requiring patient travel [83, 85]. Emerging AI‐assisted wound assessment tools, capable of automated staging of wound characteristics from standardized photographs, represent a promising development in this context; however, validation in the LVAD‐specific setting remains limited and their integration into clinical workflows should be approached with appropriate caution pending further evidence. Regular structured outpatient follow‐up remains essential for long‐term survival and prevention of device‐related complications, particularly in non‐transplant centers [94].

### 1.12 Antibiotic Therapy in LVAD‐Associated Driveline Infection

Driveline infections are predominantly caused by Gram‐positive organisms, most frequently *Staphylococcus aureus* and coagulase‐negative staphylococci, with Gram‐negative pathogens, particularly *Pseudomonas aeruginosa* , accounting for a significant proportion of cases. Candida species and other fungal organisms are encountered predominantly in deep or device‐associated infections and carry a substantially worse prognosis [21, 22]. Antibiotic therapy in LVAD‐associated DLI is stage‐directed, pathogen‐guided, and frequently long‐term, given the fundamental constraint that device removal is rarely feasible without transplantation. For prophylaxis and the general framework of infection prevention, the DESTINE group endorses the recommendations of the ISHLT consensus document [23, 41] and the EACTS expert consensus on long‐term MCS [95], to which the reader is referred for comprehensive guidance. The following positions reflect the group's opinion on practical antibiotic management in established DLI, based on accumulated clinical experience across participating centers. The initiation of chronic suppressive antimicrobial therapy should be individualized and explicitly discussed in the context of the patient's therapeutic goal (e.g., destination therapy versus bridge to transplantation). This clinical orientation fundamentally dictates the anticipated duration of support and the acceptable risk–benefit profile of prolonged antibiotic exposure. For anatomically localized and microbiologically stable infections, targeted oral suppression serves as the pragmatic first‐line strategy, with treatment durations frequently extending beyond 1 year [96]. Where oral suppression is insufficient or not feasible, intravenous antibiotic therapy should be considered and tailored to the individual resistance profile (resistogram), with agent selection guided by current susceptibility testing results. It is emphasized that suppressive therapy is a temporary strategy, not a substitute for source control. Prolonged suppression in the absence of adequate local control carries a substantive risk of resistance selection and breakthrough bacteremia [97].

## 2 Conclusion

DESTINE 2.0 establishes a refined, evidence‐based framework for the prevention and management of DLIs in LVAD patients, characterized by a simplified staging system designed to enhance clinical utility. Prevention remains the cornerstone of DLI management, and the interdependent components presented here, comprising perioperative measures, consistent DLES care, secure immobilization, appropriate antiseptic selection, and structured caregiver education, are most effective when applied as an integrated, protocol‐based continuum of care. While DLIs cannot be entirely eliminated, their frequency and severity can be substantially reduced through disciplined, standardized practice applied consistently across all care settings and throughout the full duration of LVAD support.

## Author Contributions

**Alexander M. Bernhardt:** conceptualization, methodology, writing – original draft, writing – review and editing, supervision. **Volker Lauenroth:** writing – review and editing, data curation. **Florian Mueller:** writing – review and editing. **Friedrich Kaufmann:** writing – review and editing. **Katharina Homann:** writing – review and editing. **Martina Socha:** writing – review and editing. **Thomas Schlöglhofer:** conceptualization, methodology, writing – original draft, writing – review and editing.

## Funding

The authors have nothing to report.

## Ethics Statement

The authors have nothing to report.

## Consent

The authors have nothing to report.

## Conflicts of Interest

A.M.B.: honoraria, consultancy fees, and research support from Abbott, Boston Scientific, BrioHealth, Corwave, J&J MedTech, and Shenzhen Core Medical Technology. V.L: honoraria and consultancy fees from Abbot and Medtronic. F.M. honoraria and consultancy fees from Abbot and Medtronic. T.S. consultancy/advisory roles and research grants from Abbott, Medtronic, Berlin Heart, BiVACOR, CorWave, BrioHealth Solutions, Bravida Medical, Shenzhen Core Medical Technology, and J&J MedTech. F.K., K.H. and M.S. have no conflicts to declare.

## Data Availability

The authors have nothing to report.
